# Predictive Modeling of Thoracic Radiotherapy Toxicity and the Potential Role of Serum Alpha-2-Macroglobulin

**DOI:** 10.3389/fonc.2020.01395

**Published:** 2020-08-06

**Authors:** Donata von Reibnitz, Ellen D. Yorke, Jung Hun Oh, Aditya P. Apte, Jie Yang, Hai Pham, Maria Thor, Abraham J. Wu, Martin Fleisher, Emily Gelb, Joseph O. Deasy, Andreas Rimner

**Affiliations:** ^1^Department of Radiation Oncology, Memorial Sloan Kettering Cancer Center, New York, NY, United States; ^2^Department of Medical Physics, Memorial Sloan Kettering Cancer Center, New York, NY, United States; ^3^Department of Laboratory Medicine, Memorial Sloan Kettering Cancer Center, New York, NY, United States

**Keywords:** alpha-2-macroglobulin (A2M), thoracic radiation, toxicity, radioprotection, predictive modeling

## Abstract

**Background:** To investigate the impact of alpha-2-macroglobulin (A2M), a suspected intrinsic radioprotectant, on radiation pneumonitis and esophagitis using multifactorial predictive models.

**Materials and Methods:** Baseline A2M levels were obtained for 258 patients prior to thoracic radiotherapy (RT). Dose-volume characteristics were extracted from treatment plans. Spearman's correlation (Rs) test was used to correlate clinical and dosimetric variables with toxicities. Toxicity prediction models were built using least absolute shrinkage and selection operator (LASSO) logistic regression on 1,000 bootstrapped datasets.

**Results:** Grade ≥2 esophagitis and pneumonitis developed in 61 (23.6%) and 36 (14.0%) patients, respectively. The median A2M level was 191 mg/dL (range: 94–511). Never/former/current smoker status was 47 (18.2%)/179 (69.4%)/32 (12.4%). We found a significant negative univariate correlation between baseline A2M levels and esophagitis (Rs = −0.18/*p* = 0.003) and between A2M and smoking status (Rs = 0.13/*p* = 0.04). Further significant parameters for grade ≥2 esophagitis included age (Rs = −0.32/*p* < 0.0001), chemotherapy use (Rs = 0.56/*p* < 0.0001), dose per fraction (Rs = −0.57/*p* < 0.0001), total dose (Rs = 0.35/*p* < 0.0001), and several other dosimetric variables with Rs > 0.5 (*p* < 0.0001). The only significant non-dosimetric parameter for grade ≥2 pneumonitis was sex (Rs = −0.32/*p* = 0.037) with higher risk for women. For pneumonitis D15 (lung) (Rs = 0.19/*p* = 0.006) and D45 (heart) (Rs = 0.16/*p* = 0.016) had the highest correlation. LASSO models applied on the validation data were statistically significant and resulted in areas under the receiver operating characteristic curve of 0.84 (esophagitis) and 0.78 (pneumonitis). Multivariate predictive models did not require A2M to reach maximum predictive power.

**Conclusion:** This is the first study showing a likely association of higher baseline A2M values with lower risk of radiation esophagitis and with smoking status. However, the baseline A2M level was not a significant risk factor for radiation pneumonitis.

## Introduction

Advances in radiation technology like intensity modulated radiation therapy (IMRT) and image guided RT (IGRT) have facilitated improved sparing of healthy surrounding tissues and organs. Nonetheless, radiation pneumonitis and esophagitis remain the most common dose-limiting toxicities in thoracic RT (1–6). Concurrent chemoradiation significantly increases the risk of developing pneumonitis or esophagitis compared to radiation alone (7, 8).

The reported incidence of pneumonitis after definitive thoracic RT ranges from 10 to 20%, although figures can vary greatly (3, 9–13). This is partly because pneumonitis remains a clinical diagnosis; there are no biomarkers or radiological findings that unequivocally confirm its presence. Medical intervention is required for patients with grade two or higher radiation pneumonitis, and severe cases can lead to fatal outcomes [Common Terminology Criteria for Adverse Events (CTCAE) v4.03, Supplementary Material 1]. Significant esophageal toxicity (grade 3–5) occurs in around 4% of patients with sequential chemotherapy and RT and in 18–22% of patients with concurrent chemoradiation (5, 14). Most patients experience mild symptoms like dysphagia and odynophagia while still undergoing radiation. Commonly opioids are used to control symptoms, but in severe cases, tube feeding or surgical intervention can be necessary (Supplementary Material 1).

To reduce dose-limiting toxicity in thoracic radiation, efforts have been made to adhere to normal tissue constraints derived from dose volume correlations with clinical toxicities (15). However, dose volume histograms do not fully predict clinical toxicities, as great interindividual variation remains. Intrinsic predictors of normal tissue radiation response may explain the variation and should be further analyzed.

Radioprotective agents, both natural and synthetic, can present an alternative method to prevent radiation-induced toxicity. Although this has been an active field of research for decades, only two compounds, amifostine and palifermine, are FDA-approved for the use in radiation therapy and neither is commonly used in routine thoracic RT (16–19). Another compound under investigation is alpha-2-Macroglobulin (A2M), a postulated intrinsic radioprotector. Human A2M is a glycoprotein and the largest non-immunoglobulin serum protein. In animal studies, A2M has exhibited radioprotective effects in healthy irradiated tissue. In studies with rats that underwent full body irradiation to 6.7 Gy, rats with endo- or exogenously increased levels of A2M had a higher rate of survival, regained their baseline body weight and lymphocyte count faster, and displayed normal proliferative ability of the liver tissue compared to the control groups with normal A2M levels (20–22). Suggested key mechanisms supporting the potential of A2M as a radioprotector include promoting expression of antioxidant enzymes, inhibiting fibroblast activation thus preventing fibrosis, deactivating pro-inflammatory cytokines, and enhancing DNA and cell repair mechanisms (23). Our previous study in a small cohort showed a correlation of A2M with radiation pneumonitis (9). Smoking can potentially increase A2M levels. However, literature specifically on A2M in smokers remains rare. Some studies confirmed higher A2M levels in smokers compared to non-smokers (24–26).

We investigated whether pre-treatment serum A2M levels are an independent predictive variable for the development of post-radiation toxicity in the lung and esophagus in a large cohort of patients receiving thoracic RT.

## Materials and Methods

### Patients

Clinical, laboratory, treatment and toxicity data were systematically collected in a series of thoracic RT patients between 2012 and 2016 during standard treatment and follow-up procedures. Patients were treated with either conventionally fractionated RT using 3D conformal RT (3DCRT) or intensity-modulated RT (IMRT), or with stereotactic body RT (SBRT). Patients with any prior thoracic RT were excluded. Serum samples for A2M analysis were collected at baseline prior to fraction #1 of RT. We obtained a retrospective institutional review board waiver to analyze the data. Toxicity data consist of radiation pneumonitis and esophagitis rates graded per CTCAE v4.03. Data were obtained at baseline and at routine follow-up visits every 3 months for the first 2 years.

### Alpha-2-Macroglobulin

Serum samples were taken ≤ 30 days prior to RT start, typically at the time of simulation. The mean and standard deviation between A2M measurement date and RT start date were 14 and 6 days, respectively. CLIA (Clinical Laboratory Improvement Amendments) approved A2M testing was performed at Quest Diagnostics Nichols Institute (San Juan Capistrano, CA). A2M levels were given in mg/dL; the normal range was defined as 100–280 mg/dL.

### Treatment Plans

For patients treated before 2014, treatment plans were retrieved from our in-house planning system (27). From 2014 onwards, treatments were planned in the Eclipse treatment planning system (Varian Medical Systems, Palo Alto, CA). To analyze dosimetric data, treatment plans were imported to the research platform CERR (Computational Environment for Radiological Research) for computing dosimetric variables (28). Dosimetric variables were extracted from target structures: complete esophagus for esophagitis and “lung minus gross tumor volume (GTV)” and heart for pneumonitis. Before that, plan doses were converted to equivalent dose in 2 Gy fractions (EQD2) with α/β ratio of 10 for esophagus and 3 for lung minus GTV and heart (29). As radiation esophagitis tends to develop acutely during radiotherapy, one more set of dosimetric variables for esophagus was extracted in addition to the planned doses. For these fractional variables (denoted by the prefix “f,” e.g., fmax dose) we divided the dose volume histogram (DVH) bins by the number of treatment days between the start of RT and the end of RT including weekends.

### Statistical Methods

Univariate and multivariate analyses were performed to investigate associations between toxicity (esophagitis and pneumonitis) and A2M levels, clinical, and dosimetric variables. Patients were categorized into two groups for each endpoint: non-toxicity (grade <2) and clinically significant toxicity (grade ≥2).

A Wilcoxon rank-sum test was used to find a difference in A2M expression between the two groups. Spearman's correlation (Rs) test was used to assess associations between endpoints, Dx values (minimum dose to the volume with the x% hottest dose in the organ of interest), computed from *x* = 5% to *x* = 100% in intervals of 5%, mean dose, max dose, clinical variables, and A2M. For this test CTCAE grades 0–5 were used instead of dichotomized values (grade <2 and grade ≥2).

Multivariate analysis using the least absolute shrinkage and selection operator (LASSO) logistic regression was performed using features with *p* < 0.1 that resulted from the univariate Spearman's correlation test. To avoid variable instability due to high collinearity, Pearson's correlation test was conducted among all dosimetric variables before the multivariate analysis. A cutoff of Pearson's correlation coefficient >0.75 was used to determine a relatively small group of variables for further LASSO modeling, by selecting a single variable that has the best correlation with the endpoint among a set of correlated variables after hierarchical clustering.

To rigorously verify model validity, the data were split into two groups (training data with 2/3 and validation data with 1/3 of samples). The training and validation data were balanced with cancer subtypes and outcomes. This split was performed separately for pneumonitis and esophagitis. The model building process was carried out using only the training data. Furthermore, to examine the stability of LASSO variable selection, the model building process was conducted using a bootstrapped dataset generated from the training data. Finally, the validation data were tested on the resulting model, quantified by the area under the receiver operating characteristic curve (AUC) as a function of true positive rate (sensitivity) and false positive rate (1-specificity). The final reported results represent the average performance on the validation data for predictive models built using 1,000 bootstrapped datasets.

For statistical analyses, R language (version 3.2.4), MATLAB (version 8.6.0; MathWorks. Natick, MA) and SPSS (version 24; IBM. Armonk, NY) were used.

## Results

### Patient Characteristics

In total, 258 patients were eligible for analysis. Most patients were former (*n* = 179, 69.4%) or current smokers (*n* = 32, 12.4%). One hundred and thirty-four patients (51.9%) underwent chemotherapy in addition to RT and the median total RT dose was 54 Gy (range: 27–74 Gy) for conventional fractionation and 50 Gy (range: 30–70 Gy) for SBRT. The median A2M level was 191 mg/dL (range: 94–511 mg/dL). The median follow-up was 8.9 months (range: 0.2–40.2; calculated from the start of RT). More details are available in Table 1.

**Table 1:** Patient characteristics.

**Factor**	***N***	**%**
Age [median, range]	69 (25–93) years	
Sex		
Male	122	47.3
Female	136	52.7
KPS [median, range]	90 (50–100) %	
Subgroups		
NSCLC	202	78.3
SCLC	17	6.6
Thymoma	8	3.1
Mesothelioma	25	9.7
Lung metastases (other primary)	6	2.3
Smoking history		
Never	47	18.2
Former	179	69.4
Current	32	12.4
Pack-years (former/current smokers) [median, range]	37 (1–204) years	
Alpha-2-macroglobulin [median, range]	191 (94–511) mg/dL	
Chemotherapy timing		
Concurrent	60	23.2
Sequential	74	28.7
No chemotherapy	124	48.1
RT total dose [median, range]		
Conventional RT	54 (27–74) Gy	
SBRT	50 (30–70) Gy	
Follow-up time (from start of RT) [median, range]	8.9 (0.2–40.2) months	
Time to toxicity [median]	1.0 months (esophagitis), 3.6 months (pneumonitis)	

### Toxicities

Fifty-third patients (20.5%) experienced grade two and eight (3.1%) grade three radiation esophagitis. No grade four or five esophagitis was observed. Median time to development of esophagitis was 0.85 months after the start of RT (range: 0.2–6.47 months). Grade two radiation pneumonitis developed in 26 patients (10.1%), grade three in nine (3.5%) and grade four in one patient (0.4%). No grade five pneumonitis was observed. Median time to development of pneumonitis was 4.7 months after the start of RT (range: 1.3–8.1 months).

Of the patients who developed grade ≥2 esophagitis, 8 (13.1%) were never, 43 (70.5%) former and 10 (16.4%) current smokers whereas in pneumonitis 9 (25%) were never, 24 (66.7%) former and 3 (8.3%) current smokers.

### Univariate Analysis

#### Alpha-2-Macroglobulin

A significant correlation between baseline A2M values and esophagitis was found (Rs = −0.18/*p* = 0.003). Using a Wilcoxon rank-sum test, we found that patients with grade <2 had significantly higher baseline serum A2M levels than patients with grade ≥2 esophagitis (*p* = 0.015) as shown in Table 2. No statistically significant difference was found between baseline A2M levels and grade ≥2 pneumonitis (*p* = 0.84).

**Table 2:** Comparison of mean A2M serum levels [mg/dL] between grade <2 and ≥2 esophagitis and pneumonitis.

**Toxicity**		**Grade 0 or 1**	**Grade 2+**	***p*-value**
Esophagitis	*N*	197	61	0.015
	Mean A2M	208.9	190.4	
Pneumonitis	*N*	222	36	0.837
	Mean A2M	204.1	207.0	

*P-value was calculated using Wilcoxon rank-sum test*.

A trend between smoking status and A2M levels was observed. Current smokers had higher levels (217.3 mg/dl) compared to former (207.3 mg/dl) and never smokers (185.4 mg/dl), and former smokers had higher levels compared to never smokers. The A2M level had a significant correlation with a status of former/current smoker when compared to never smokers (Rs = 0.13/*p* = 0.04).

#### Clinical Factors

Among standard clinical variables, the following variables showed significant correlations with grade ≥2 esophagitis: age (Rs = −0.32/*p* < 0.0001), fraction number (Rs = 0.64/*p* < 0.0001), treatment days (Rs = 0.60/*p* < 0.0001), chemotherapy use (Rs = 0.56/*p* < 0.0001), dose per fraction (Rs = −0.57/*p* < 0.0001), and total dose (Rs = 0.35/*p* < 0.0001), whereas for grade ≥2 pneumonitis, the only significant clinical variable was sex (Rs = −0.32/*p* = 0.037) with a higher risk for women.

#### Dosimetric Factors

**Figure 1:**
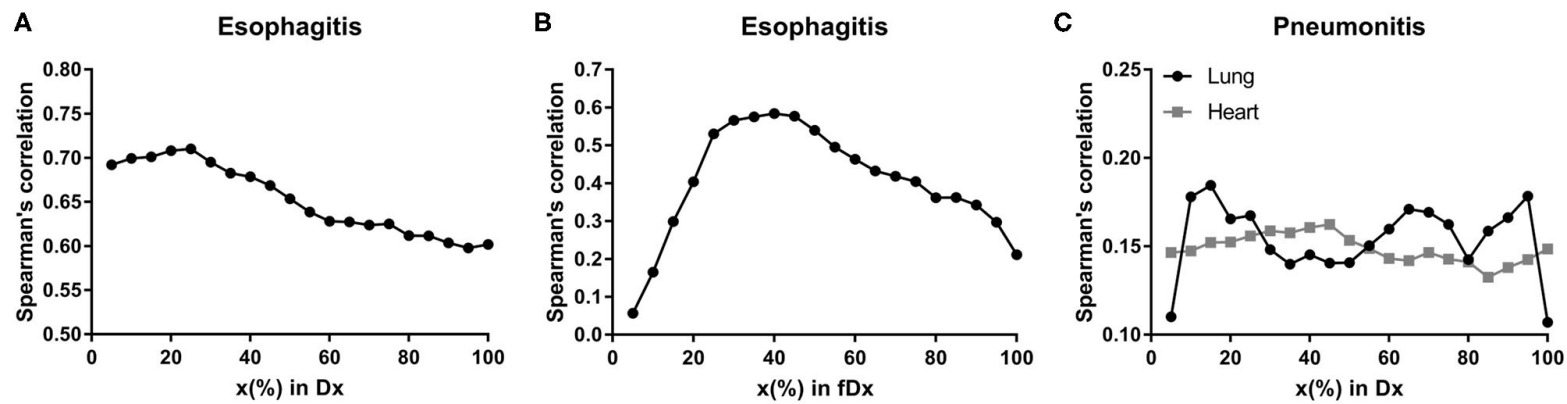
Spearman's correlation coefficients. Spearman's correlation coefficients between radiation-induced injuries (≥ grade 2) and Dx in esophagus for **(A)** esophagitis, fDx in esophagus for **(B)** esophagitis, and Dx in lung and heart for **(C)** pneumonitis.

Spearman's correlation test between dosimetric variables in esophagus and esophagitis showed that all variables had Rs > 0.60 (*p* < 0.0001) as shown in Figure 1A. For the fractional dose, fD40 was the highest correlated variable (Rs = 0.58/*p* < 0.0001) as shown in Figure 1B.

D15 (Rs = 0.19/*p* = 0.006) in lung and D45 (Rs = 0.16/*p* = 0.016) in heart were assessed as the highest correlated variables with pneumonitis for each organ (Figure 1C). Maximum dose in heart was also significantly correlated with Rs = 0.14 (*p* = 0.043).

### Multivariate Analysis and Validation Testing

Hierarchical clustering coupled with Pearson's correlation test using training data was performed on dosimetric variables of each organ to measure variable similarity. Many dosimetric variables were highly correlated (Supplementary Material 2). Using a threshold of 0.75 in Pearson's correlation the variable with the highest Rs value for the endpoint among the variables in each cluster was selected. Clinical variables with *p* < 0.1 in the univariate analysis and dosimetric variables left after the clustering test were used in the LASSO logistic regression: D25, D40, D50, D65, D85, fD10, fD25, fD35 in esophagus, age, total dose, and A2M for esophagitis; D10, D15, D65, D95 in lung, D20, D45, max dose in heart and sex for pneumonitis. For both endpoints, treatment days, SBRT (yes/no) and chemotherapy (yes/no) were used. Two variables including dose per fraction and number of fractions were excluded due to their high correlation with the number of treatment days.

**Figure 2:**
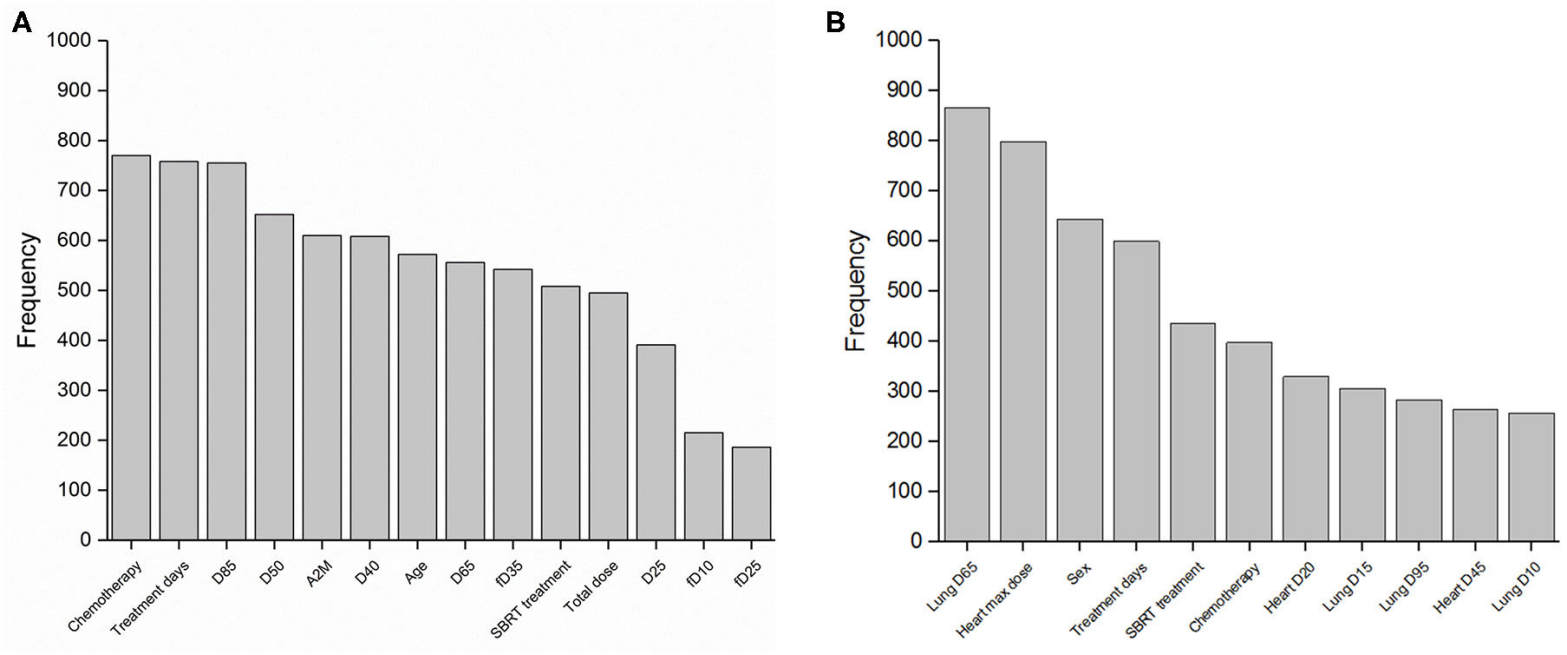
Features in predictive models. Frequency of occurrence of each feature used in 1,000 predictive models for **(A)** esophagitis and **(B)** pneumonitis.

**Figure 3:**
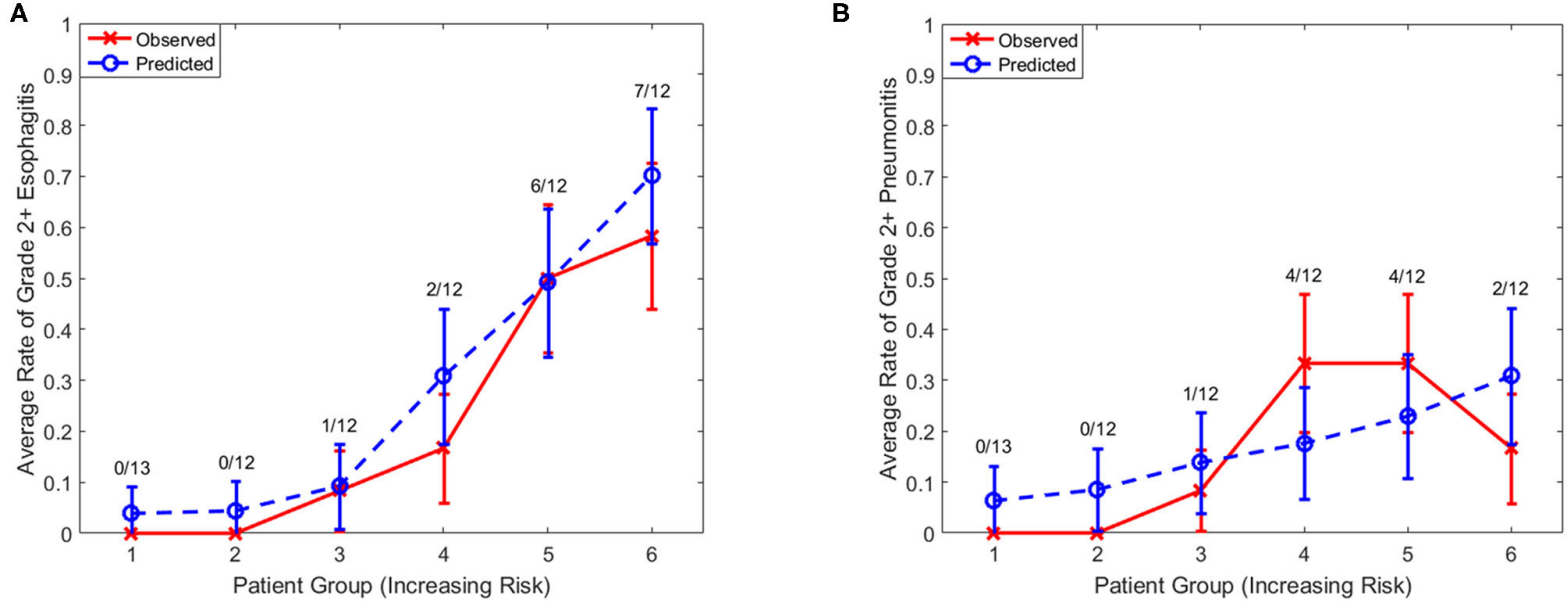
Observed and predicted incidence. Comparison of observed and predicted incidence on validation data (1/3 of samples) for **(A)** esophagitis and **(B)** pneumonitis. Numerator, number of events in each bin; Denominator, number of samples in each bin.

LASSO logistic regression models were trained using bootstrapped datasets generated from training data and were tested on the validation data, resulting in an average AUC of 0.84 (standard deviation [SD] = 0.03) and 0.78 (SD = 0.06) for esophagitis and pneumonitis, respectively. Additional modeling was performed for esophagitis without A2M resulting in the same average AUC (0.84). This appears to be due to more significant dosimetric and clinical variables used in the modeling. To assess the importance of features, the frequency of occurrence of each feature during the model building process was counted (Figure 2). For the esophagitis model, chemotherapy and treatment days were most frequently selected with 770 and 758 times from 1,000 different models, respectively. It is worth noting that A2M was selected 610 times, implying its likely association with esophagitis. For the pneumonitis model, D65 in lung and max dose in heart were most frequently selected with 865 and 798 times, respectively. Patients were sorted based on predicted outcomes on the validation data and grouped into six equal bins with one being the lowest risk group and six being the highest risk group. When comparing observed and predicted incidence, we found a high conformity of both endpoints, meaning that the predictive models are highly robust (Figure 3). Final predictive models built using all training data are shown in Table 3. Interestingly, a variable of treatment days (between the start of RT and the end of RT including weekends) was selected in both models.

**Table 3:** Final predictive models for esophagitis and pneumonitis.

**Variable**	**Coefficient**	**STD**	**Odds ratio**	**95% CI**	
**Esophagitis model**					
D25	0.012	0.026	1.012	0.962	1.064
D40	0.036	0.022	1.037	0.993	1.083
Treatment days	0.048	0.026	1.049	0.997	1.105
Constant	−3.880	0.725	0.021	0.005	0.086
**Pneumonitis model**					
D65 in lung	0.252	0.146	1.286	0.967	1.711
Max dose in heart	0.015	0.008	1.015	0.999	1.032
Treatment days	0.024	0.020	1.024	0.986	1.064
Constant	−3.824	0.793	0.022	0.005	0.103

*CI, confidence interval; STD, standard deviation*.

**Figure 4:**
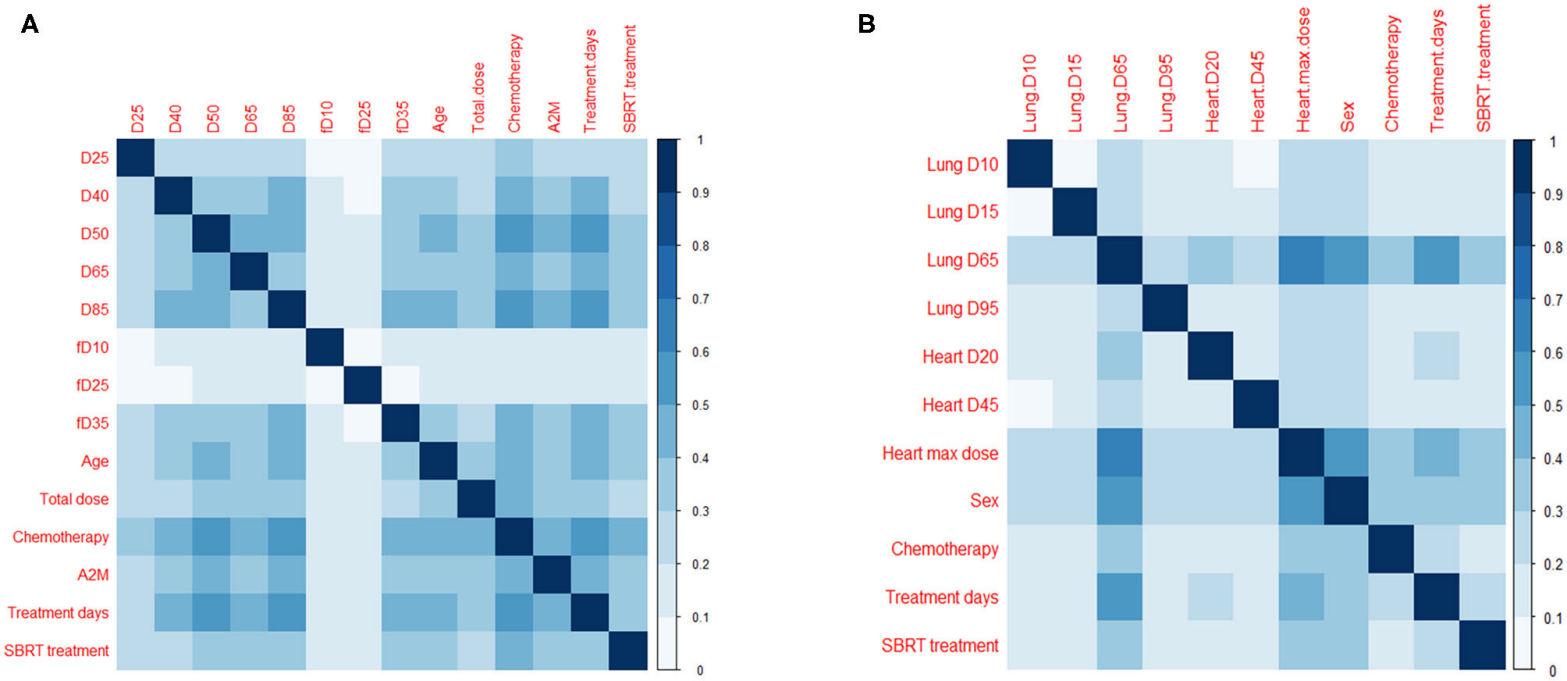
Pairs of features in predictive models. Frequency of occurrence of a pair of features (divided by 1,000) used in 1,000 predictive models for **(A)** esophagitis and **(B)** pneumonitis.

In addition, the frequency of occurrence of each pair of features used in the LASSO logistic regression model was investigated (Figure 4), which provides the information of interaction effects of features in the predictive model.

## Discussion

Taking into account the multifactorial etiology of radiation toxicity (30), it is essential to look at different predictive factors in the development of lung and esophageal injury after RT. Dosimetric parameters are most commonly included in predictive models but biological and genetic determinants are also under investigation (9, 11, 30–37). In our analysis, we focused on dose-volume metrics, age, chemotherapy, and other clinical variables in addition to the intrinsic radioprotectant A2M.

As we identified in our correlative analysis, baseline serum A2M levels appear to be influenced by patients' smoking status. Former and current smokers displayed higher A2M values than patients that had never smoked. Active and former smoking has been associated with lower rates of grade ≥3 radiation pneumonitis compared to never smokers in patients with NSCLC after 3DCRT or IMRT (38). The effect of smoking on the immune system has been studied extensively. Paradoxically, smoking results in immunosuppression as well as aggravated autoimmunity. Altered levels of inflammatory cytokines like TNF-α, IFN-γ, IL-1β, IL-6, IL-8, IL-10, and others have been reported in healthy smokers (39–42). A possible explanation for the connection between active smoking and a lower risk for esophagitis or pneumonitis is that long-term cigarette smoking leads to an increased immune response in the lung and surrounding tissues due to the damage it inflicts on the lung parenchyma. Although the mechanisms resulting in normal tissue injury after RT are still under investigation, the release of reactive oxygen species (ROS) as well as proinflammatory and profibrotic cytokines is thought to have a central role in the process (30). Higher baseline levels of acute-phase proteins like A2M may have a protective effect on the irradiated tissue by binding proinflammatory and profibrotic cytokines, thus reducing the acute cytokine toxicity, and inducing an upregulation of antioxidant enzymes like manganese superoxide dismutase (MnSOD) (23, 30).

Our study suggests that there may be an association between natural pre-treatment baseline levels of the intrinsic radioprotectant serum A2M in patients with thoracic malignancies and an increased risk of developing radiation esophagitis. This association reached univariate statistical significance for esophagitis, but not for the pneumonitis endpoint. This finding indicates that higher levels of A2M may have a protective effect in patients undergoing thoracic RT. The high selection frequency of A2M in the model building process confirms our primary univariate analyses implying a likely correlation of A2M levels with esophagitis rates. Factors that have repeatedly shown significant correlation with esophagitis include V40–V60 (6, 43–46) (Vx: percentage volume receiving at least x Gy), mean esophageal dose (47–49), as well as sequential and especially concurrent chemoradiation in comparison to RT alone (5, 6, 50–53).

For pneumonitis, we validated the correlation with radiation dose received by the heart. Different lung dose volumes (V5–V40 and mean dose in lung) have been found to predict the development of pneumonitis (7, 10, 54). In addition, the dose received by the heart during thoracic radiation seems to be an accurate predictor (55, 56). The best fitting predictive model reported by Huang et al. included D10 (heart), D35 (lung) and max dose (lung), and had an AUC of 0.72 (55). Although the ideal dosimetric variable(s) for predicting pneumonitis across all patient subgroups may not yet be known, it is evident that heart doses are an essential part of any model built for this cause. Though we could not confirm the impact of A2M on pneumonitis with our data, a correlation between them has been previously described (9, 56). We may have been limited by the lower incidence of grade ≥2 pneumonitis (14.0%) and the low rate of current smokers in our patient cohort. In the previously published study on A2M and pneumonitis, pneumonitis rates were between 19 and 35%. The variable of sex was found to be correlated with pneumonitis in univariate analysis and had the third-highest frequency in 1,000 model runs (Figure 2B), consistent with another study (57). However, its role as a risk factor for pneumonitis is controversial (58).

While all patients had A2M collected within 30 days prior to the start of RT and toxicity data were systematically prospectively graded per our clinical standard, caution is warranted regarding the interpretation of these results. In particular, including a factor like chemotherapy in predictive models should be considered carefully as different regimens, doses and timings, depending on the patient population, make it a very heterogeneous variable. Similarly, the patient cohort we studied was diverse regarding diagnosis and treatment. Although requirements for eligibility included no prior RT, patients underwent different modes of RT (3DCRT/SBRT/IMRT) which may have an impact on the toxicity profile. Furthermore, smoking status as a variable was not evenly distributed. Given its proposed link to serum A2M levels, this could be a cause for certain discrepancies in our results. A2M levels in humans reported in previous studies range on average between 100 and 450 mg/dL with a mean of around 215 mg/dL. Age and gender are known to influence this value with females generally showing around 20% higher levels than same-aged males (59, 60). Given the lack of data thus far concerning other factors that may influence intrinsic A2M levels, further analyses with serum levels recorded immediately prior to and while receiving RT are necessary.

In summary, the analysis of our institutional dataset has produced predictive models for both esophagitis and pneumonitis. Although the addition of A2M did not increase the predictive power of multivariate predictive models, this is the first report on the possible association of higher levels of A2M with a lower risk of radiation esophagitis and with smoking, warranting further investigation and independent validation.

## Data Availability Statement

The datasets generated for this study are available on request to the corresponding author.

## Ethics Statement

The studies involving human participants were reviewed and approved by Memorial Sloan Kettering Cancer Center. The patients/participants provided their written informed consent to participate in this study.

## Author Contributions

JD, JO, AR, DR, AW, and EY contributed to conception and design of the study. MF, EG, AR, and DR acquired and organized the data. AA, JD, JO, HP, MT, and JY analyzed the data and performed the statistical analysis. JO, AR, and DR drafted the manuscript. All authors contributed to the critical revision and approved the submission of the manuscript.

## Conflict of Interest

JD has research contracts with Varian Medical Systems and Philips. AR has grants from Varian Medical Systems, Boehringer Ingelheim, Pfizer, Astrazeneca, Merck, and personal fees from Astrazeneca, Merck, Cybrexa, MoreHealth, and ResearchtoPractice as well as non-financial support from Philips/Elekta. AW has a grant from CivaTech Oncology, personal fees from AstraZeneca, and a travel grant from AlphaTau Medical. The remaining authors declare that the research was conducted in the absence of any commercial or financial relationships that could be construed as a potential conflict of interest. The handling editor declared a past collaboration with two of the authors MT and JD.
