# Supplementary material for: Predictive Modeling of Thoracic Radiotherapy Toxicity and the Potential Role of Serum Alpha-2-Macroglobulin
Source: Front Oncol. 2020 Aug 6;10:1395. doi: 10.3389/fonc.2020.01395 (PMC7423838; doi:10.3389/fonc.2020.01395)
Supplement: Supplementary file 1 [file Data_Sheet_1.PDF]

Supplementary Material 1. CTCAE v4.03 grading for pneumonitis and esophagitis.

ADVERSE EVENT	**Pneumonitis**<br>*Definition: A disorder characterized by inflammation focally or diffusely affecting the lung parenchyma*	**Esophagitis**<br>*Definition: A disorder characterized by inflammation of the esophageal wall.*
**Grade 1**	Asymptomatic; clinical or diagnostic observations only; intervention not indicated	Asymptomatic; clinical or diagnostic observations only; intervention not indicated
**Grade 2**	Symptomatic; medical intervention indicated; limiting instrumental ADL	Symptomatic; altered eating/swallowing; oral supplements indicated
**Grade 3**	Severe symptoms; limiting self-care ADL; oxygen indicated	Severely altered eating/swallowing; tube feeding, TPN or hospitalization indicated
**Grade 4**	Life-threatening respiratory compromise; urgent intervention indicated (e.g., tracheotomy or intubation)	Life-threatening consequences; urgent operative intervention indicated
**Grade 5**	Death	Death
