# Supplementary figures and images for: Predictive Modeling of Thoracic Radiotherapy Toxicity and the Potential Role of Serum Alpha-2-Macroglobulin
Source: Front Oncol. 2020 Aug 6;10:1395. doi: 10.3389/fonc.2020.01395 (PMC7423838; doi:10.3389/fonc.2020.01395)

(A)

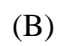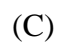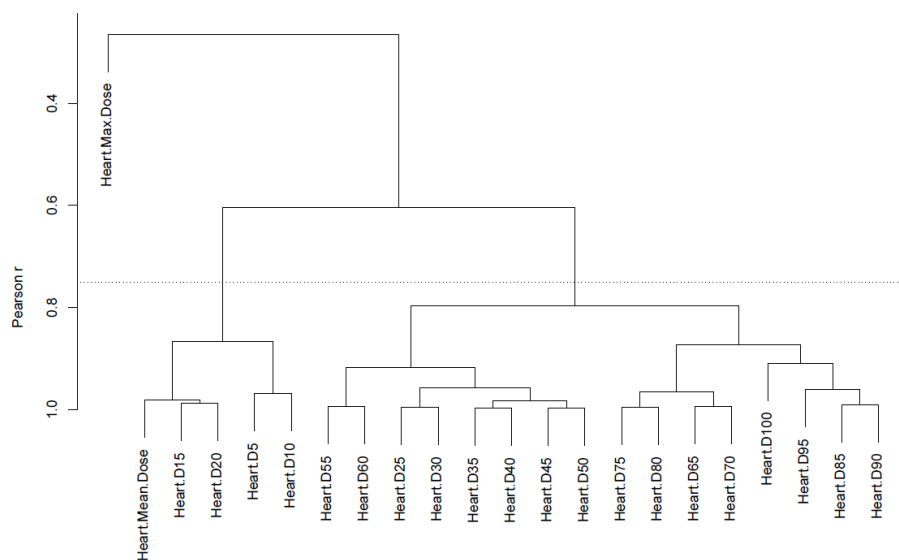

Supplement: Supplementary file 2 [file Data_Sheet_2.PDF]
